# Editorial: Cytokines, and biomarkers involved in the immunomodulation of pediatric cancers

**DOI:** 10.3389/fimmu.2025.1713006

**Published:** 2025-11-13

**Authors:** Alessandro Poggi, Gabriella Pietra, Ignazio Caruana, Francesca Romana Mariotti, Bruno Azzarone

**Affiliations:** 1 Integrated Oncology Therapies, San Martino Hospital (IRCCS), Genova, Italy; 2 Department of Experimental Medicine (DIMES), University of Genova, Genova, Italy; 3 UO Pathology and Experimental Immunology, IRCCS Ospedale Policlinico San Martino, Genova, Italy; 4 Department of Pediatrics, Haematology, Oncology, Stem Cell Transplantation and Cell Therapy, University Hospital Würzburg, Würzburg, Germany; 5 Tumor Immunology Unit, IRCCS Bambino Gesù Children's Hospital, Rome, Italy

**Keywords:** pediatric cancer, tumor microenvironment, immune response, soluble molecules, combination therapies, immunotherapies

Childhood cancers emerge primarily from genetic predisposition, in contrast to adult malignancies, where stochastic DNA errors accumulate with age and environment (1). Furthermore, pediatric tumors often originate from embryonic-derived cells, with biology and microenvironment iterations that differ substantially from the adult cancers (2). However, in both pediatric and adult cancers, tumor microenvironment (TME) plays a key role. This dynamic ecosystem—composed of stromal cells, immune cells, vasculature, and extracellular matrix (ECM) components as well as a plethora of soluble molecules/elements including growth factors, cytokines, chemokines, hormones, enzymes, and extracellular vesicles—critically shapes tumor progression and influences the effectiveness of therapies (3, 4). This complexity is further increased by the presence of tumor cells in a different state of maturation/differentiation together with genetic and metabolic heterogeneity and, consequently, heterogeneity of the TME itself (4, 5). However, the TME is a highly dynamic place in which therapeutic tools influence its composition and trigger the generation of resistant tumor cells implied in the refractory/relapsing and possibly tumor host cell death (5). Resistance, relapse, and collateral tissue effects often emerge from this interplay. Neuroblastoma (NB) offers a paradigmatic example of how stromal and immune components govern disease course (6). Maggi et al. have faced this matter and dissected the NB TME, highlighting the heterogeneity of tumor-infiltrating T cells and their crosstalk with NB cells and cancer-associated fibroblasts (CAFs), both of which demonstrate a certain heterogeneity and ability to influence, through the production of several cytokines, the antitumor function of T cells. Suppressive players like myeloid-derived suppressor cells (MDSC), tumor associated macrophages (TAM) and tumor-associated neutrophils (TAN) are increasingly recognized as central to pediatric tumor immunology (7, 8). Evidence across NB, sarcomas, and other childhood malignancies shows that these cells blunt T and NK cell response through cell-to-cell contact and the secretion of several soluble mediators such as TGF-β1, MIF, IDO, arginase-2 and soluble MICA and B7-H6. This immunosuppressive axis appears as a recurring theme in the pediatric TME, underscoring the need for therapeutic strategies able to reprogram/target these compartments (Maggi et al.). In order to decode the role and relevance of NK cell-mediated anti-tumor response in NB, Di Matteo et al. identified two subsets of primary tumors - mature CD105+CD133- and undifferentiated CD105-CD133+ - that differ in susceptibility to NK-mediated killing. Both the tumor subsets expressed the therapeutic target disialoganglioside 2 (GD2) reinforcing the clinical impact of anti-GD2 therapies such as Dinutuximab beta Apeiron, which trigger NK cell-mediated antibody-dependent cellular cytotoxicity (ADCC), as well as GD2-redirected chimeric antigen receptor therapies. Beyond NB, Pellegrino et al. review the immunosuppressive role of the IGF1/IGF1R axis in childhood cancers. By acting on both tumor cells and regulatory lymphocytes, IGF1 signaling dampens adaptive and innate immunity. Targeting this pathway reduces immunosuppressive populations such as regulatory T cells, TAMs, and MDSCs, while boosting effector T cells, NK cells, and professional antigen-presenting cells. In this study, the authors also reported the hypothesis that autologous cell immunotherapy with IGF1R antisense strategies may further induce tumor stress, activating immunogenic cell death (ICD) (9–13).

The activation of a strong antitumor response associated with the ICD is the topic of the manuscript from Ye et al. This is a good example of the possibility of triggering this effect using oncolytic vaccinia virus (VACV) co-expressing interleukin 2 (IL2) and tumor antigens. In murine models, these viruses triggered robust antigen-specific T cell responses, outperforming constructs expressing IL-2 or tumor antigen alone. However, translating these approaches to children requires nuance. The pediatric immune system, unlike the adult population, is still developing, with thymic T cell selection, NK repertoire editing, and immune checkpoint regulation in flux (14–17). Moreover, growth-related pathways such as IGF1/IGF1R are essential to organ development in children, adding complexity to therapeutic targeting (18, 19). These developmental layers must be carefully integrated into therapeutic design to avoid unintended autoimmunity or growth impairment while maximizing anti-tumor efficacy.

In conclusion, the studies collected in this Research Topic emphasize the unique immunobiology of pediatric tumors. From the immunosuppressive myeloid and stroma cells that dominate the TME to NK- and T-cell-mediated immunity, to the modulation of pathways like IGFR and the induction of ICD, it is clear that immunomodulation in children follows rules distinct from adults. A deeper understanding of cytokines and biomarkers in this setting will lead to designing safe, effective, and durable immunotherapies for childhood malignancies.
